# Growth hormone secretion is diminished and tightly controlled in humans enriched for familial longevity

**DOI:** 10.1111/acel.12519

**Published:** 2016-09-07

**Authors:** Evie van der Spoel, Steffy W. Jansen, Abimbola A. Akintola, Bart E. Ballieux, Christa M. Cobbaert, P. Eline Slagboom, Gerard Jan Blauw, Rudi G. J. Westendorp, Hanno Pijl, Ferdinand Roelfsema, Diana van Heemst

**Affiliations:** ^1^ Section Gerontology and Geriatrics Department of Internal Medicine Leiden University Medical Center Leiden The Netherlands; ^2^ Department of Clinical Chemistry and Laboratory Medicine Leiden University Medical Center Leiden The Netherlands; ^3^ Section Molecular Epidemiology Department of Medical Statistics Leiden University Medical Center Leiden The Netherlands; ^4^ Department of Public Health and Center of Healthy Aging University of Copenhagen Copenhagen Denmark; ^5^ Section Endocrinology Department of Internal Medicine Leiden University Medical Center Leiden The Netherlands

**Keywords:** approximate entropy, familial longevity, growth hormone, hormone secretion, human, IGF‐1

## Abstract

Reduced growth hormone (GH) signaling has been consistently associated with increased health and lifespan in various mouse models. Here, we assessed GH secretion and its control in relation with human familial longevity. We frequently sampled blood over 24 h in 19 middle‐aged offspring of long‐living families from the Leiden Longevity Study together with 18 of their partners as controls. Circulating GH concentrations were measured every 10 min and insulin‐like growth factor 1 (IGF‐1) and insulin‐like growth factor binding protein 3 (IGFBP3) every 4 h. Using deconvolution analysis, we found that 24‐h total GH secretion was 28% lower (*P* = 0.04) in offspring [172 (128–216) mU L^−1^] compared with controls [238 (193–284) mU L^−1^]. We used approximate entropy (ApEn) to quantify the strength of feedback/feedforward control of GH secretion. ApEn was lower (*P* = 0.001) in offspring [0.45 (0.39–0.53)] compared with controls [0.66 (0.56–0.77)], indicating tighter control of GH secretion. No significant differences were observed in circulating levels of IGF‐1 and IGFBP3 between offspring and controls. In conclusion, GH secretion in human familial longevity is characterized by diminished secretion rate and more tight control. These data imply that the highly conserved GH signaling pathway, which has been linked to longevity in animal models, is also associated with human longevity.

## Introduction

Genetic disruption of the insulin/insulin‐like growth factor 1 (IGF‐1) signaling (IIS) pathway can delay aging and promote longevity in a wide variety of species (Longo & Finch, 2003). In mammalian species, growth hormone (GH) plays a pivotal role in the regulation of the IIS pathway and mutations affecting GH action have consistently been shown to alter lifespan (Bartke *et al*., 2013). Increased longevity in mice can be induced by mutations that result in GH deficiency, including the Prop‐1 and Pit‐1 mutations that cause a combined GH, prolactin, and thyroid‐stimulating hormone deficiency, and by deletion of the GH‐releasing hormone receptor (Brown‐Borg *et al*., 1996; Flurkey *et al*., 2001). Likewise, mutations resulting in GH resistance, notably deletion of the GH receptor, were also found to increase longevity (Coschigano *et al*., 2000). Accordingly, transgenic mice that overexpress GH are short‐lived and show signs of accelerated aging (Wolf *et al*., 1993). Also in humans, patients with active acromegaly, who have excessive pituitary GH secretion, were found to have a reduced life expectancy (Orme *et al*., 1998). The results from studies on the association of mutations in the GH pathway that lead to dwarfism in humans with lifespan are contradictory (Sattler, 2013). While Laron syndrome dwarfs with GH receptor gene mutations were found to have relatively long lifespans with reduced risks for cancer and diabetes, patients with untreated GH deficiency had relatively short lifespans (Laron, 2002; Besson *et al*., 2003; Guevara‐Aguirre *et al*., 2011).

However, little is known about how more subtle differences in GH/IGF‐1 secretion would affect human longevity. Interestingly, female centenarians were found to be enriched for rare mutations causing slight IGF‐1 resistance and resulting in a somewhat smaller stature (Suh *et al*., 2008). Likewise, we previously observed that a combination of polymorphisms in the GH/IIS pathway, linked to smaller stature in female octogenarians, was associated with better survival in old age (van Heemst *et al*., 2005). However, to the best of our knowledge, no study has assessed the association of human longevity with GH secretion.

GH secretion by somatotrophic cells in the anterior lobe of the pituitary gland is stimulated by growth hormone‐releasing hormone (GHRH) and inhibited by somatostatin, both produced by the hypothalamus. GH exerts its functions by binding to GH receptors located on tissue target cells. A key function of GH is to stimulate production of IGF‐1 by the liver, which subsequently inhibits GH secretion via negative feedback. Circulating IGF‐1 is mostly bound to binding proteins of which insulin‐like growth factor binding protein 3 (IGFBP3) is the most abundant. The IGF‐1/IGFBP3 molar ratio is considered an indicator of IGF‐1 bioavailability. In humans, many other tissues besides the liver express GH receptors indicating that GH may exert effects independent from IGF‐1 (Florini *et al*., 1996; de Mello‐Coelho *et al*., 1998; Arce *et al*., 2013). GH is secreted in a basal (nonpulsatile) and pulsatile mode. The feedback control by IGF‐1, together with the feedforward control by GHRH and somatostatin, tightly regulates GH secretion (Giustina & Veldhuis, 1998). The strength of these control signals can be estimated mathematically via calculation of the approximate entropy (ApEn) (Veldhuis *et al*., 2001). Both GH secretion and the strength of the feedforward and/or feedback signals were found to be negatively influenced by age and BMI (Zadik *et al*., 1985; Veldhuis *et al*., 2011). Interestingly, women had higher 24‐h serum GH concentrations and higher ApEn values than men, indicating weaker control of GH secretion (Pincus *et al*., 1996). Furthermore, GH secretion is strongly associated with sleep, with the major GH pulse generally occurring shortly after sleep onset (Takahash *et al*., 1968).

To identify determinants of human longevity, the Leiden Longevity Study (LLS) included offspring of long‐lived families that are enriched for exceptional longevity and partners thereof, serving as a control group. Indeed, offspring were found to have less age‐related diseases and reduced mortality compared with controls (Westendorp *et al*., 2009). Previously, no differences were observed between offspring and controls in circulating IGF‐1 concentrations (Rozing *et al*., 2009). However, the magnitude and control of GH secretion have not yet been studied in human familial longevity. Therefore, we aim in this study to compare GH secretion parameters and the strength of GH secretion control signals between offspring of long‐lived families and age‐matched controls.

## Results

### Group characteristics

The group characteristics of offspring and controls are presented in Table 1. Participants were selected on the basis of the age of their parents. Consequently, the parents of the offspring were significantly older (*P* = 0.01) than those of the controls. The groups of offspring and controls were similar in age, height, body composition, fasting serum glucose and insulin levels, and sleep parameters. Also the distribution of men and women in both groups was similar.

**Table 1:** Group characteristics of offspring of long‐lived families and controls

	Offspring *n* = 19	Controls *n* = 18	*P*‐value
Male *n* (%)	10 (52.6)	10 (55.6)	0.86
Age (years)^a^	65.7 (5.6)	64.6 (4.9)	0.52
BMI (kg m^−2^)^b^	24.8 (22.7–29.9)	25.1 (22.1–27.7)	0.83
Height (cm)^b^	175 (165–180)	175 (167–182)	0.73
Waist circumference (cm)^b^	94 (82–101)	94 (83–98)	0.75
Fasting glucose (mmol L^−1^)^a^	4.9 (0.7)	4.8 (0.4)	0.65
Fasting insulin (mU L^−1^)	4.6 (3.8–8.0)	5.9 (3.8–7.8)	0.92
Average hours of sleep (h)	7.4 (7.0–7.9)	7.4 (6.9–7.8)	0.81
Chronotype^c^	3.0 (2.5–3.5)	3.1 (2.6–3.6)	0.68
Mean age of parents (years)	88.0 (82.5–93.5)	80.3 (74.6–84.1)	**0.001**

Unless indicated otherwise, data are presented as median with interquartile ranges.

a Data are presented as mean with standard deviation.

b Data were not available for one male control subject due to technical problems.

c Scores ranging from 1 (extreme early type) to 7 (extreme late type).

Bold values indicate *P* ≤ 0.05.

### GH concentration profiles over 24 h

Individual GH concentration profiles were first assessed by visual inspection. As illustrated in Fig. S1 (Supporting information), which comprises the 24‐h GH concentration profiles of all 37 participants, we observed a wide variation between individual 24‐h GH profiles. Moreover, due to the pulsatile manner in which GH is secreted, GH concentrations within individual time series vary strongly over 24 h. Therefore, deconvolution analysis was applied to compare specific features of GH secretion between groups.

### GH secretion parameters

Table 2 shows that the offspring of long‐lived families had a mean (95% CI) total GH secretion over 24 h of 172 (128–216) mU L^−1^. This was significantly lower (*P* = 0.04) compared with that of controls (238 (193–284) mU L^−1^). The geometric mean (95% CI) basal GH secretion in offspring (14.5 (9.8–21.5) mU L^−1^) was also lower (*P* = 0.03) compared with that of controls (26.9 (17.9–40.4) mU L^−1^). The pulsatile GH secretion was not significantly different between groups. Results did not change after additional adjustments for BMI or waist circumference (data not shown). Slow half‐life, the number of pulses, and the interpulse regularity (Weibull gamma) were similar between offspring and controls. Similar differences as found between offspring and controls were also observed in men and women separately (data not shown).

**Table 2:** GH secretion parameters in offspring of long‐lived families and controls

	Offspring *n* = 19	Controls *n* = 18	*P*‐value
Slow half‐life (minutes)	16.2 (14.3–18.0)	15.2 (13.3–17.1)	0.46
Total secretion (mU L^−1^ per 24 h)	172 (128–216)	238 (193–284)	**0.04**
Basal secretion (mU L^−1^ per 24 h)^a^	14.5 (9.8–21.5)	26.9 (17.9–40.4)	**0.03**
Pulsatile secretion (mU L^−1^ per 24 h)	151 (113–188)	191 (152–230)	0.14
Number of pulses (per 24 h)^a^	13.7 (12.1–15.5)	13.8 (12.1–15.7)	0.97
Interpulse regularity γ (unitless)	1.3 (1.2–1.5)	1.5 (1.3–1.7)	0.26

Unless indicated otherwise, data are presented as mean with 95% confidence interval.

a Data are presented as geometric mean with 95% confidence interval. Analyzed by linear mixed model adjusted for sex and age.

### Approximate entropy

**Figure 1:**
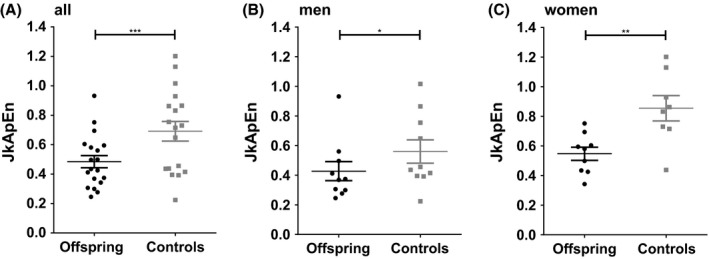
Control of GH secretion in offspring and controls, with lower jack‐knifed approximate entropy (JkApEn) indicating tighter control. JkApEn of GH secretion of offspring and controls in all subjects (A), men (B) and women (C). Lines indicate mean and standard error of the mean. Analyzed by linear mixed model adjusted for sex and age (A) or only age (B and C).*P ≤ 0.05, **P ≤ 0.01, ***P ≤ 0.001.

Offspring of long‐lived families had a significantly lower (*P* = 0.001) Jack‐knifed approximate entropy (JkApEn) than controls (Fig. 1). Figure 1 shows the individual JkApEn for all participants with the (unadjusted) mean with standard error. When analyses were adjusted for age and sex, the geometric mean (95% CI) JkApEn was 0.45 (0.39–0.53) for offspring and 0.66 (0.56–0.77) for controls (*P* = 0.001). The differences found between offspring and controls were also observed in men and women separately. Male offspring had a geometric mean (95% CI) JkApEn of 0.38 (0.29–0.48) and male controls of 0.53 (0.42–0.68) (*P* = 0.05). The geometric mean (95% CI) JkApEn of female offspring was significantly lower (*P* = 0.005) than that of female controls [0.53 (0.44–0.65) vs. 0.82 (0.67–1.01)]. Results did not materially change after additional adjustments for BMI or waist circumference (data not shown).

### IGF‐1 and IGFBP3

Geometric mean (95% CI) circulating levels of IGF‐1 were 14.2 (12.6–15.9) nmol L^−1^ in the offspring and 16.0 (14.2–18.0) nmol L^−1^ in controls, which was not significantly different (*P* = 0.14). Mean IGFBP3 was similar in both groups. The mean (95% CI) IGF‐1/IGFBP3 molar ratio was also not significantly different (*P* = 0.10) in offspring compared with controls [0.128 (0.116–0.140) vs. 0.142 (0.130–0.155)].

## Discussion

The two main findings of this study are that GH secretion is lower and more tightly controlled in subjects enriched for familial longevity compared with age‐matched controls.

The observed association between reduced GH secretion and human familial longevity is in line with experimental studies in mice, which found that reduced GH action resulted in extended health and lifespans (Brown‐Borg *et al*., 1996; Coschigano *et al*., 2000; Flurkey *et al*., 2001). Our results implicate the highly conserved GH/IGF‐1 signaling pathway, which has been linked to delayed aging and longevity in numerous animal models, also in human longevity. The observed differences in GH secretion between offspring and controls can probably not be explained by a faster clearance of GH from the blood, as the slow half‐life was comparable between groups. Previous studies found that high approximate entropy (ApEn) values are indicative for reduced strength of feedforward and/or feedback signals on GH secretion. ApEn values were elevated in patients with GH deficiency and in patients with an inactivating defect of the GHRH receptor gene, compared to healthy individuals (Roelfsema *et al*., 2001, 2002). Accordingly, we observed low values of ApEn in the offspring, which are indicative of tighter control.

Various mechanisms may underlie the observed reduction in GH secretion in the offspring, including (i) less stimulatory feedforward drive by GHRH, (ii) increased inhibition by somatostatin, and (iii) increased negative feedback of IGF‐1. Veldhuis *et al*. (2001) showed that reduced ApEn values can also be explained by any of these three occurrences. Our current data do not allow us to discriminate between these possibilities.

Although somatostatin and GHRH are found in the circulation, these were not measured as it is unknown to what extent these reflect leakage from the brain, or peripheral production. We did measure circulating levels of IGF‐1 and IGFBP3, and calculated IGF‐1/IGFBP3 molar ratios. The IGF‐1/IGFBP3 molar ratio was not higher in the offspring than in the controls. However, other important IGF‐1 binding proteins were not measured; thus, we cannot exclude the possibility that differences in IGF‐1 bioavailability between groups may exist. Altered GH secretion can also be caused by regulators of GH secretion other than somatostatin, GHRH, and IGF‐1. Certain neuropeptides and neurotransmitters also influence GH secretion, such as ghrelin from the stomach, which directly stimulates the release of GH by the pituitary (Giustina & Veldhuis, 1998; Dimaraki & Jaffe, 2006).

Different explanations exist for the observed association of diminished GH secretion and familial longevity. A first possibility is that GH is associated with longevity via a reduction of circulating IGF‐1. In contrast to long‐living model organisms in which besides GH action, IGF‐1 levels are reduced, IGF‐1 levels were not significantly different between groups, although these tended to be somewhat lower in the offspring. Possibly, our study was underpowered to detect subtle differences in circulating IGF‐1 between groups. However, also in a much larger sample of offspring and controls, we previously did not observe differences in circulating IGF‐1, nor in stature (Rozing *et al*., 2009). Circulating IGF‐1 is predominantly produced by the liver. However, many tissues produce paracrine IGF‐1 that does not contribute to circulating IGF‐1. Interestingly, in Ames dwarf mice with reduced GH secretion, brain IGF‐1 levels were elevated compared to control mice (Sun *et al*., 2005). In the current study, data on tissue‐specific IGF‐1 are lacking. Therefore, the possibility that paracrine IGF‐1 is crucial in terms of longevity control could not be addressed. It is also possible that GH is associated with longevity independent of IGF‐1. In support of this hypothesis, the impact of disrupting GH signaling on longevity is larger than the impact of disrupting IGF‐1 signaling or events downstream from IGF‐1 receptors in experimental mice (Bartke, 2011). Also in humans, many tissues besides the liver express GH receptors, and the association between reduced GH and human familial longevity might be caused by direct effects of GH on these tissues (Florini *et al*., 1996; de Mello‐Coelho *et al*., 1998; Arce *et al*., 2013). Lastly, it is possible that GH secretion is not causally related to longevity, but rather a pleiotropic side effect of upstream regulators that influence longevity via other mechanisms. For example, in addition to inhibiting GH secretion, somatostatin influences the secretion of other hormones and was found to inhibit cell proliferation and induce apoptosis (Weckbecker *et al*., 2003). Also GHRH was found to have pleotropic effects in the periphery, mainly on tissue regeneration (Kiaris *et al*., 2011).

A strength of our study is that we sampled blood every 10 min during 24 h. Therefore, we could study the somatotropic axis in detail. We performed our study in the LLS, in which families are included based on two proband nonagenarian siblings. A consequence of the LLS study design is that the age range of the offspring varied from 52 to 76 years and GH profiles could not be obtained at young age. Another limitation of this study design is that not every offspring might be enriched for longevity, as possibly not all inherited the favorable predisposition for longevity of their long‐lived parent. This could have diluted potential differences between offspring and controls. It is also a limitation that this study was performed with a relatively small sample size.

To conclude, we report for the first time that GH secretion is more tightly controlled in the offspring of long‐lived families than in controls. We hypothesize that the offspring are therefore more efficient in regulating the magnitude and the timing of GH secretion. Our data strengthen the hypothesis that GH/IGF‐1 signaling is a conserved mechanism implicated in mammalian longevity. We hypothesize that pleiotropic and possibly tissue‐specific effects of reduced GH secretion may favor human longevity. Future research should focus on dissecting the mechanisms via which reduced GH is associated with human longevity.

## Experimental procedures

### Study population

As described previously in more detail, the Leiden Longevity Study (LLS) comprises 421 families with at least two long‐lived Caucasian siblings fulfilling the age criteria (men ≥89 years and women ≥91 years) without selection on health or demographics (Westendorp *et al*., 2009). In the Switchbox Leiden Study (protocol P11.116), we included 20 offspring of nonagenarian LLS participants together with 18 partners of the offspring as environmental and age‐matched controls (Jansen *et al*., 2015). All participants were middle‐aged (52–76 years), had a stable body mass index (BMI) between 18 and 34 kg m^−2^, and women were postmenopausal. Exclusion criteria were having chronic renal, hepatic or endocrine disease, or using medication known to influence lipolysis, thyroid function, glucose metabolism, GH or IGF‐1 secretion and/or any other hormonal axis. Moreover, participants were excluded based on the presence of fasting plasma glucose >7 mmol L^−1^, recent transmeridian flight, smoking addiction, or extreme diet therapies. To be able to safely perform the 24‐h blood sampling, other exclusion criteria were difficulties to insert and maintain an intravenous catheter, anemia (hemoglobin <7.1 mmol L^−1^), and blood donation within the last 2 months. Based on information obtained via telephone questioning, controls with a nonagenarian parent who had one or more nonagenarian siblings were also excluded. The Switchbox Leiden Study was approved by the Medical Ethical Committee of the Leiden University Medical Centre and performed according to the Helsinki declaration. All participants gave written informed consent for participation. For the current study, we excluded one female offspring from the analyses based on a combination of features that suggest a subtle form of GH resistance. Her pulsatile GH secretion (539 mU L^−1^) deviated more than three standard deviations from the mean pulsatile secretion of the women included (193 mU L^−1^), while her height (158 cm) was relatively short compared to the mean height of the women included (165 cm).

### Clinical protocol

Full details on the 24‐h blood sampling procedure have been described previously (Akintola *et al*., 2015). In short, a catheter was placed in a vein of the forearm of the nondominant hand of the participant. Blood sampling started around 09:00 h and every 10 min, 3.2 mL of blood was collected. All participants were sampled in the same research room. The participants received standardized feeding at three fixed times during the day (between 09:00 h and 10:00 h, 12:00 h and 13:00 h, and 18:00 h and 19:00 h), each consisting of 600 kcal Nutridrink (Nutricia Advanced Medical Nutrition Zoetermeer, The Netherlands). No naps were allowed during the day, and lights were switched off for approximately 9 h (circa between 23:00 h and 08:00 h). Height and weight were measured in the research center. Body mass index (BMI) was calculated as weight (in kilograms) divided by the square of height (in meters). Waist circumference was measured with a measuring tape midway between the uppermost border of the iliac crest and the lower border of the costal margin.

### Assays

All measurements were performed at the Department of Clinical Chemistry and Laboratory Medicine of the Leiden University Medical Centre in The Netherlands, which is accredited according to CCKL (National Coordination Committee for Quality Assurance for Health Care Laboratories in The Netherlands). Laboratory measurements were performed with fully automated equipment and diagnostics from Roche Diagnostics (Almere, The Netherlands) and Siemens Healthcare diagnostics (The Hague, The Netherlands). Human growth hormone (hGH) with a molecular mass of 22 kDa was measured in serum samples using Siemens reagents (catalog number L2KGRH2) and an IMMULITE^®^ 2000 Xpi Immunoassay system (Siemens Healthcare diagnostics). The detection limit was 0.15 mU L^−1^, and the interassay coefficient of variation (CV) ranged between 5.4% at 5.43 mU L^−1^ and 7.2% at 25.0 mU L^−1^. The reference range for fasting GH is 0.15–7.25 mU L^−1^. All samples were measured with reagents from the same lot numbers, and for each participant, all samples (one time series) were measured in the same batch on the same day. IGF‐1 (catalog number IS‐3900) and IGFBP3 (catalog number IS‐4400) were measured in six plasma EDTA samples with 4 h intervals for each participant using an iSYS Immunoassay system of ImmunoDiagnostic Systems (IDS GMBH, Frankfurt am Main, Germany). The CV ranges for IGF‐1 and IGFBP3 were 1.4–1.8% and 6.3–7.3%, respectively. Insulin (catalog number L2KIN2) and glucose (catalog number 11876899216) were analyzed in a fasting morning (around 8:30 h) serum sample; insulin (CV ranged between 3.2 and 7.7%) using an IMMULITE^®^ 2000 Xpi Immunoassay system (Siemens Healthcare diagnostics) and glucose (CV ranged between 0.9 and 3.0%) using Hitachi Modular P800 (Roche Diagnostics). In the Netherlands, hGH and IGF‐1 test results are routinely harmonized using a national harmonization protocol and a harmonization serum pool as described elsewhere (Ross *et al*., 2014). This means that all hGH and IGF‐1 results reported in this study are multiplied with 1.02 and 1.13, respectively.

### Deconvolution analysis

The 24‐h GH concentration profiles were analyzed by validated deconvolution analysis (Liu *et al*., 2009). By deconvolution analysis, a GH concentration profile is decomposed into underlying secretory bursts, basal secretion, elimination of previously secreted GH, and random experimental variability. The algorithm in the software program MATLAB (the Mathworks, Inc., Natick, MA) first detrends the data and normalizes concentrations to numbers within the interval 0 to 1. Thereafter, successive potential pulse‐time sets, each containing one fewer burst, were created by a smoothing process. Finally, a maximum‐likelihood expectation deconvolution method estimated all secretion and elimination rates simultaneously for each candidate pulse‐time set. Outcome parameters of main interest are basal (nonpulsatile) secretion, pulsatile secretion, the sum of basal and pulsatile secretion (total secretion), number of pulses per 24 h (secretory‐burst frequency), interpulse regularity (Weibull gamma), and slow half‐life. Fast half‐life was fixed to 3.5 min, and slow half‐life was estimated as unknown variable between 8 and 22 min.

### Approximate entropy

Approximate entropy (ApEn) is a scale‐ and model‐independent statistic that quantifies the regularity of consecutive time‐series data (Pincus, 1991). ApEn has high sensitivity and specificity (both >90%) for analysis of hormone concentration measurements over 24 h. Low ApEn values imply that the sequence of time‐series data is regular and that it contains many repetitive patterns, such as a sinus wave. High ApEn values indicate greater irregularity and randomness. For hormonal data, ApEn provides a direct barometer for the strength of the feedback system. In neuro‐endocrine time‐series of a length of 50–300 data points, *m* (window length) = 1 is preferred, and for lengths *N* ≥ 60, *r* (criterion of similarity) should be set to the predetermined value of 20% of the standard deviation (SD) of the individual subject time series (Pincus *et al*., 1999). Therefore, normalized ApEn parameters of *m* = 1 and *r* = 20% of the SD of the individual subject time series were used. Additionally, we calculated the Jack‐knifed ApEn (JkApEn), which is a rigorous and objective cross‐validation test that gives less bias in smaller samples than regular ApEn and it is more applicable for hormone data (Meyfroidt *et al*., 2010).

### Chronotype and sleep

Data on sleep parameters were obtained using the Pittsburgh Sleep Quality Index questionnaire (Buysse *et al*., 1989). Chronotype was assessed using the Munich Chronotype questionnaire, with scores ranging from 1 (extreme early type) to 7 (extreme late type) (Roenneberg *et al*., 2003; Zavada *et al*., 2005).

### Statistical analysis

Descriptive statistics were used to summarize the characteristics of study groups. The nonparametric Median Test was used to assess differences between offspring and controls in the variables that were not normally distributed. Independent‐samples *t*‐test was used to assess differences between offspring and controls in variables that were normally distributed. GH secretion parameters were compared between offspring and controls using linear regression adjusted for sex and age. Not normally distributed parameters were logarithmic transformed prior to analysis and are presented as geometric means with 95% confidence intervals. *P* ≤ 0.05 was considered statistically significant. All statistical analyses were performed with SPSS for Windows, version 20 (SPSS, Chicago, IL, USA). Graphs were made using GraphPad Prism version 6 (GraphPad, San Diego, CA, USA).

## Funding

This research is funded by the European Commission project Switchbox (FP7, Health‐F2‐2010‐259772). Evie van der Spoel is supported by a personal PhD grant from the Leiden University Medical Center.

## Author contributions

DvH, FR, RW, and HP designed the study. SWJ, AAA, and EvdS acquired the data. EvdS, SWJ, and FR analyzed the data. EvdS and DvH drafted the manuscript. EvdS, SWJ, AAA, BB, CC, PES, GJB, RW, HP, FR, and DvH interpreted the data and critically revised the manuscript for important intellectual content.

## Conflict of interest

The authors have no conflict of interest.

## Supporting information


**Fig. S1** 24‐h GH concentration profiles of all 37 participants.

## References

[acel12519-bib-0001] Akintola AA , Jansen SW , Wilde RB , Hultzer G , Rodenburg R , van Heemst D (2015) A simple and versatile method for frequent 24 h blood sample collection in healthy older adults. MethodsX. 2, 33–38. 26150969 10.1016/j.mex.2014.12.003 PMC4487324

[acel12519-bib-0002] Arce VM , Devesa P , Devesa J (2013) Role of growth hormone (GH) in the treatment on neural diseases: from neuroprotection to neural repair. Neurosci. Res. 76, 179–186. 23602740 10.1016/j.neures.2013.03.014

[acel12519-bib-0003] Bartke A (2011) Pleiotropic effects of growth hormone signaling in aging. Trends Endocrinol. Metab. 22, 437–442. 21852148 10.1016/j.tem.2011.07.004 PMC4337825

[acel12519-bib-0004] Bartke A , Sun LY , Longo V (2013) Somatotropic signaling: trade‐offs between growth, reproductive development, and longevity. Physiol. Rev. 93, 571–598. 23589828 10.1152/physrev.00006.2012 PMC3768106

[acel12519-bib-0005] Besson A , Salemi S , Gallati S , Jenal A , Horn R , Mullis PS , Mullis PE (2003) Reduced longevity in untreated patients with isolated growth hormone deficiency. J. Clin. Endocrinol. Metab. 88, 3664–3667. 12915652 10.1210/jc.2002-021938

[acel12519-bib-0006] Brown‐Borg HM , Borg KE , Meliska CJ , Bartke A (1996) Dwarf mice and the ageing process. Nature 384, 33. 10.1038/384033a0 8900272

[acel12519-bib-0007] Buysse DJ , Reynolds CF III , Monk TH , Berman SR , Kupfer DJ (1989) The Pittsburgh Sleep Quality Index: a new instrument for psychiatric practice and research. Psychiatry Res. 28, 193–213. 2748771 10.1016/0165-1781(89)90047-4

[acel12519-bib-0008] Coschigano KT , Clemmons D , Bellush LL , Kopchick JJ (2000) Assessment of growth parameters and life span of GHR/BP gene‐disrupted mice. Endocrinology 141, 2608–2613. 10875265 10.1210/endo.141.7.7586

[acel12519-bib-0009] Dimaraki EV , Jaffe CA (2006) Role of endogenous ghrelin in growth hormone secretion, appetite regulation and metabolism. Rev. Endocr. Metab. Disord. 7, 237–249. 17195943 10.1007/s11154-006-9022-0

[acel12519-bib-0010] Florini JR , Ewton DZ , Coolican SA (1996) Growth hormone and the insulin‐like growth factor system in myogenesis. Endocr. Rev. 17, 481–517. 8897022 10.1210/edrv-17-5-481

[acel12519-bib-0011] Flurkey K , Papaconstantinou J , Miller RA , Harrison DE (2001) Lifespan extension and delayed immune and collagen aging in mutant mice with defects in growth hormone production. Proc. Natl Acad. Sci. USA 98, 6736–6741. 11371619 10.1073/pnas.111158898 PMC34422

[acel12519-bib-0012] Giustina A , Veldhuis JD (1998) Pathophysiology of the neuroregulation of growth hormone secretion in experimental animals and the human. Endocr. Rev. 19, 717–797. 9861545 10.1210/edrv.19.6.0353

[acel12519-bib-0013] Guevara‐Aguirre J , Balasubramanian P , Guevara‐Aguirre M , Wei M , Madia F , Cheng CW , Hwang D , Martin‐Montalvo A , Saavedra J , Ingles S , de Cabo R , Cohen P , Longo VD (2011) Growth hormone receptor deficiency is associated with a major reduction in pro‐aging signaling, cancer, and diabetes in humans. Sci. Transl. Med. 3, 70ra13. 10.1126/scitranslmed.3001845 PMC3357623 21325617

[acel12519-bib-0014] van Heemst D , Beekman M , Mooijaart SP , Heijmans BT , Brandt BW , Zwaan BJ , Slagboom PE , Westendorp RG (2005) Reduced insulin/IGF‐1 signalling and human longevity. Aging Cell 4, 79–85. 15771611 10.1111/j.1474-9728.2005.00148.x

[acel12519-bib-0015] Jansen SW , Akintola AA , Roelfsema F , van der Spoel E , Cobbaert CM , Ballieux BE , Egri P , Kvarta‐Papp Z , Gereben B , Fekete C , Slagboom PE , van der Grond J , Demeneix BA , Pijl H , Westendorp RGJ , van Heemst D (2015) Human longevity is characterised by high thyroid stimulating hormone secretion without altered energy metabolism. Sci. Rep. 5, 11525. 26089239 10.1038/srep11525 PMC4473605

[acel12519-bib-0016] Kiaris H , Chatzistamou I , Papavassiliou AG , Schally AV (2011) Growth hormone‐releasing hormone: not only a neurohormone. Trends Endocrinol. Metab. 22, 311–317. 21530304 10.1016/j.tem.2011.03.006

[acel12519-bib-0017] Laron Z (2002) Effects of growth hormone and insulin‐like growth factor 1 deficiency on ageing and longevity. Novartis Found. Symp. 242, 125–137. 11855684

[acel12519-bib-0018] Liu PY , Keenan DM , Kok P , Padmanabhan V , O'Byrne KT , Veldhuis JD (2009) Sensitivity and specificity of pulse detection using a new deconvolution method. Am. J. Phys. Endocrinol. Metab. 297, E538–E544. 10.1152/ajpendo.00071.2009 PMC2724108 19531646

[acel12519-bib-0019] Longo VD , Finch CE (2003) Evolutionary medicine: from dwarf model systems to healthy centenarians? Science 299, 1342–1346. 12610293 10.1126/science.1077991

[acel12519-bib-0020] de Mello‐Coelho V , Gagnerault MC , Souberbielle JC , Strasburger CJ , Savino W , Dardenne M , Postel‐Vinay MC (1998) Growth hormone and its receptor are expressed in human thymic cells. Endocrinology 139, 3837–3842. 9724037 10.1210/endo.139.9.6199

[acel12519-bib-0021] Meyfroidt G , Keenan DM , Wang X , Wouters PJ , Veldhuis JD , Van den Berghe G (2010) Dynamic characteristics of blood glucose time series during the course of critical illness: effects of intensive insulin therapy and relative association with mortality. Crit. Care Med. 38, 1021–1029. 20124887 10.1097/CCM.0b013e3181cf710e

[acel12519-bib-0022] Orme SM , Mcnally RJQ , Cartwright RA , Belchetz PE (1998) Mortality and cancer incidence in acromegaly: a retrospective cohort study. J. Clin. Endocrinol. Metab. 83, 2730–2734. 9709939 10.1210/jcem.83.8.5007

[acel12519-bib-0023] Pincus SM (1991) Approximate entropy as a measure of system‐complexity. Proc. Natl Acad. Sci. USA 88, 2297–2301. 11607165 10.1073/pnas.88.6.2297 PMC51218

[acel12519-bib-0024] Pincus SM , Gevers EF , Robinson ICAF , VandenBerg G , Roelfsema F , Hartman ML , Veldhuis JD (1996) Females secrete growth hormone with more process irregularity than males in both humans and rats. Am. J. Phys. Endocrinol. Metab. 270, E107–E115. 10.1152/ajpendo.1996.270.1.E107 8772482

[acel12519-bib-0025] Pincus SM , Hartman ML , Roelfsema F , Thorner MO , Veldhuis JD (1999) Hormone pulsatility discrimination via coarse and short time sampling. Am. J. Phys. Endocrinol. Metab. 277, E948–E957. 10.1152/ajpendo.1999.277.5.E948 10567024

[acel12519-bib-0026] Roelfsema F , Biermasz NR , Veldman RG , Veldhuis JD , Frolich M , Stokvis‐Brantsma WH , Wit JM (2001) Growth hormone (GH) secretion in patients with an inactivating defect of the GH‐releasing hormone (GHRH) receptor is pulsatile: evidence for a role for non‐GHRH inputs into the generation of GH pulses. J. Clin. Endocrinol. Metab. 86, 2459–2464. 11397840 10.1210/jcem.86.6.7536

[acel12519-bib-0027] Roelfsema F , Biermasz NR , Veldhuis JD (2002) Pulsatile, nyctohemeral and entropic characteristics of GH secretion in adult GH‐deficient patients: selectively decreased pulsatile release and increased secretory disorderliness with preservation of diurnal timing and gender distinctions. Clin. Endocrinol. 56, 79–87. 10.1046/j.0300-0664.2001.01433.x 11849250

[acel12519-bib-0028] Roenneberg T , Wirz‐Justice A , Merrow M (2003) Life between clocks: daily temporal patterns of human chronotypes. J. Biol. Rhythms 18, 80–90. 12568247 10.1177/0748730402239679

[acel12519-bib-0029] Ross HA , Lentjes EW , Menheere PM , Sweep CG (2014) Harmonization of growth hormone measurement results: the empirical approach. Clin. Chim. Acta 432, 72–76. 24508625 10.1016/j.cca.2014.01.008

[acel12519-bib-0030] Rozing MP , Westendorp RGJ , Frolich M , de Craen AJM , Beekman M , Heijmans BT , Mooijaart SP , Blauw GJ , Slagboom PE , van Heemst D (2009) Human insulin/IGF‐1 and familial longevity at middle age. Aging‐Us. 1, 714–722. 10.18632/aging.100071 PMC2806046 20157552

[acel12519-bib-0031] Sattler FR (2013) Growth hormone in the aging male. Best Pract. Res. Clin. Endocrinol. Metab. 27, 541–555. 24054930 10.1016/j.beem.2013.05.003 PMC3940699

[acel12519-bib-0032] Suh Y , Atzmon G , Cho MO , Hwang D , Liu B , Leahy DJ , Barzilai N , Cohen P (2008) Functionally significant insulin‐like growth factor I receptor mutations in centenarians. Proc. Natl Acad. Sci. USA 105, 3438–3442. 18316725 10.1073/pnas.0705467105 PMC2265137

[acel12519-bib-0033] Sun LY , Al‐Regaiey K , Masternak MM , Wang J , Bartke A (2005) Local expression of GH and IGF‐1 in the hippocampus of GH‐deficient long‐lived mice. Neurobiol. Aging 26, 929–937. 15718052 10.1016/j.neurobiolaging.2004.07.010

[acel12519-bib-0034] Takahash Y , Kipnis DM , Daughada WH (1968) Growth hormone secretion during sleep. J. Clin. Invest. 47, 2079–2790. 5675428 10.1172/JCI105893 PMC297368

[acel12519-bib-0035] Veldhuis JD , Straume M , Iranmanesh A , Mulligan T , Jaffe C , Barkan A , Johnson ML , Pincus S (2001) Secretory process regularity monitors neuroendocrine feedback and feedforward signaling strength in humans. Am. J. Phys. Regul. Integr. Comp. Phys. 280, R721–R729. 10.1152/ajpregu.2001.280.3.R721 11171650

[acel12519-bib-0036] Veldhuis JD , Roelfsema F , Keenan DM , Pincus S (2011) Gender, age, body mass index, and IGF‐I individually and jointly determine distinct GH dynamics: analyses in one hundred healthy adults. J. Clin. Endocrinol. Metab. 96, 115–121. 20926525 10.1210/jc.2010-1669 PMC3038492

[acel12519-bib-0037] Weckbecker G , Lewis I , Albert R , Schmid HA , Hoyer D , Bruns C (2003) Opportunities in somatostatin research: biological, chemical and therapeutic aspects. Nat Rev Drug Discov. 2, 999–1017. 14654798 10.1038/nrd1255

[acel12519-bib-0038] Westendorp RGJ , van Heemst D , Rozing MP , Frolich M , Mooijaart SP , Blauw GJ , Beekman M , Heijmans BT , de Craen AJM , Slagboom PE (2009) Nonagenarian siblings and their offspring display lower risk of mortality and morbidity than sporadic nonagenarians: the Leiden Longevity Study. J. Am. Geriatr. Soc. 57, 1634–1637. 19682117 10.1111/j.1532-5415.2009.02381.x

[acel12519-bib-0039] Wolf E , Kahnt E , Ehrlein J , Hermanns W , Brem G , Wanke R (1993) Effects of long‐term elevated serum levels of growth‐hormone on life expectancy of mice – lessons from transgenic animal‐models. Mech. Ageing Dev. 68, 71–87. 8350664 10.1016/0047-6374(93)90141-d

[acel12519-bib-0040] Zadik Z , Chalew SA , Mccarter RJ , Meistas M , Kowarski AA (1985) The influence of age on the 24‐hour integrated concentration of growth‐hormone in normal individuals. J. Clin. Endocrinol. Metab. 60, 513–516. 3972964 10.1210/jcem-60-3-513

[acel12519-bib-0041] Zavada A , Gordijn MC , Beersma DG , Daan S , Roenneberg T (2005) Comparison of the Munich chronotype questionnaire with the Horne‐Ostberg's morningness‐eveningness score. Chronobiol. Int. 22, 267–278. 16021843 10.1081/cbi-200053536

