# Supplementary material for: Growth hormone secretion is diminished and tightly controlled in humans enriched for familial longevity
Source: Aging Cell. 2016 Sep 7;15(6):1126–31. doi: 10.1111/acel.12519 (PMC6398524; doi:10.1111/acel.12519)
Supplement: Supplementary file 1 — Fig. S1 24‐h GH concentration profiles of all 37 participants. [file ACEL-15-1126-s001.pdf]

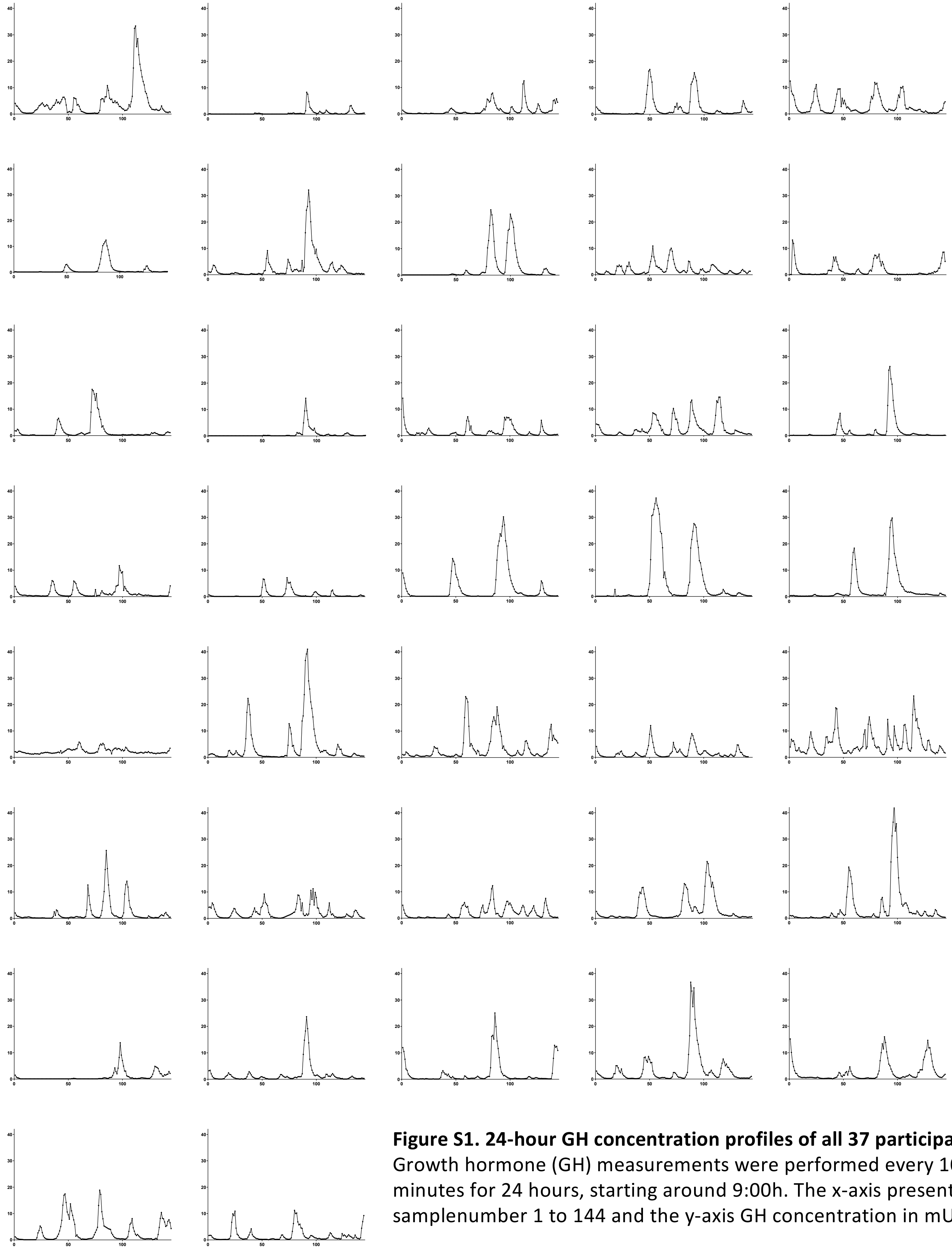

Figure S1. 24-hour GH concentration profiles of all 37 participants
Growth hormone (GH) measurements were performed every 10 minutes for 24 hours, starting around 9:00h. The x-axis presents samplenumber 1 to 144 and the y-axis GH concentration in mU/L.
